# Applications of Single-Molecule Vibrational Spectroscopic Techniques for the Structural Investigation of Amyloid Oligomers

**DOI:** 10.3390/molecules27196448

**Published:** 2022-09-30

**Authors:** Katrin Ha Phuong Vu, Gerhard Heinrich Blankenburg, Leonardo Lesser-Rojas, Chia-Fu Chou

**Affiliations:** 1 Nanoscience and Technology Program, Taiwan International Graduate Program, Academia Sinica, Taipei 11529, Taiwan; 2 Department of Engineering and System Science, National Tsing Hua University, Hsinchu 300044, Taiwan; 3 Institute of Physics, Academia Sinica, Taipei 11529, Taiwan; 4 Department of Physics, National Taiwan University, Taipei 10617, Taiwan; 5 Research Center for Atomic, Nuclear and Molecular Sciences, University of Costa Rica, San Pedro de Montes de Oca, San José 2060, Costa Rica; 6 School of Physics, University of Costa Rica, San Pedro de Montes de Oca, San José 2060, Costa Rica; 7 Research Center for Applied Sciences, Academia Sinica, Taipei 11529, Taiwan

**Keywords:** vibrational spectroscopy, amyloid oligomers, amyloid intermediates, protein structure, single molecule

## Abstract

Amyloid oligomeric species, formed during misfolding processes, are believed to play a major role in neurodegenerative and metabolic diseases. Deepening the knowledge about the structure of amyloid intermediates and their aggregation pathways is essential in understanding the underlying mechanisms of misfolding and cytotoxicity. However, structural investigations are challenging due to the low abundance and heterogeneity of those metastable intermediate species. Single-molecule techniques have the potential to overcome these difficulties. This review aims to report some of the recent advances and applications of vibrational spectroscopic techniques for the structural analysis of amyloid oligomers, with special focus on single-molecule studies.

## 1. Introduction

**Figure 1:**
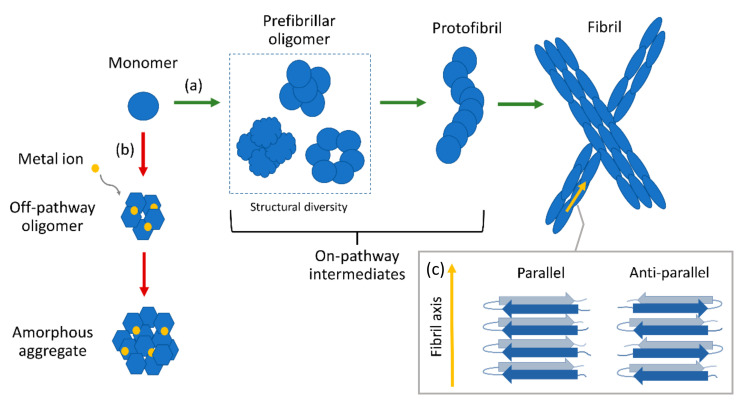
Schematic illustration of amyloid aggregation. (**a**) On-pathway mechanism: native monomers misfold and undergo conformational change to form prefibrillar oligomers, protofibrils, and mature fibrils. (**b**) Alternative pathway induced by metal ions: monomers form off-pathway oligomers which do not end up as fibrils but amorphous aggregates. (**c**) Schematic illustration of parallel and anti-parallel cross-β structure. Amyloid fibrils have parallel cross-β conformation.

The formation of fibrillar peptide and protein aggregates and their deposition within and around cells is a hallmark of numerous neurodegenerative and metabolic diseases, ranging from Alzheimer’s and Parkinson’s diseases to type II diabetes and dialysis-related amyloidosis [1]. Those so-called amyloid fibrils are structurally defined by stacked β-strands running perpendicular to the long fibril axis (cross-β structure) (Figure 1c). A range of amyloid-forming species, such as amyloid-β (Aβ), α-synuclein, tau, and the islet amyloid polypeptide (IAPP), are intrinsically disordered in their native states [1]. During the ordinary (“on-pathway”) process of aggregation, those peptides undergo structural reorganization to form metastable intermediate species called oligomers, which further self-assemble into protofibrils and finally into mature fibrils, as illustrated in Figure 1a. An increasing body of evidence suggests that it is not the mature fibrils, but rather the oligomeric intermediates that exhibit higher toxicity [2,3,4]. For instance, studies have shown that Aβ oligomers and tau oligomers can cause synaptic disfunction [5,6,7,8,9,10] and impair membrane integrity [11,12,13,14]. Similarly, α-synuclein [15] and IAPP [16,17] oligomers were found to exert toxicity through membrane disruption. In addition, it has been shown that metal ions, such as Cu^2+^, Zn^2+^, or Fe^3+^, can alter the aggregation pathway of amyloids [18,19,20,21]. For instance, Zn^2+^ has been reported to bind to Aβ peptides and induce the formation of off-pathway oligomers, which do not end up as fibrils but as large, amorphous aggregates (Figure 1b) [22,23]. The properties and structures of toxic amyloid oligomeric species are of immense interest in order to understand amyloid disease and amyloid formation, yet the oligomeric structures are not studied in as much detail as the mature fibrillar forms. It is due to their heterogeneity in conformation and size, their transient nature, and their low abundance that those intermediate species are difficult to characterize with conventional structure determination methods, such as X-ray diffraction (XRD) or nuclear magnetic resonance (NMR) spectroscopy. X-ray diffraction has substantially contributed to the determination of amyloid fibril structure by first demonstrating the characteristic cross-β conformation [24]. However, most amyloid intermediate species cannot be studied using XRD, as this technique requires the peptide samples to be of high purity and crystallized, which usually is not possible for amyloid oligomers. A brief introduction to common spectroscopic methods for protein structure determination, as well as a comparison between them, is given in the next section. We want to emphasize that most conventional techniques only provide information averaged over an ensemble of molecules and thus are typically unable to extract information about individual species within the diverse populations of amyloid intermediates. The aim of this review is to highlight some of the recent advances in vibrational spectroscopic techniques and tools which to this date have been used for the structural investigation of amyloid intermediate species while putting emphasis on single molecule techniques, as those are best suited to overcoming the issue of heterogeneity and low abundance of amyloid intermediates.

## 2. General Introduction and Comparison of Vibrational Spectroscopy and Other Common Spectroscopic Methods

Vibrational spectroscopy, such as Fourier transform infrared spectroscopy (FTIR) and Raman spectroscopy, are commonly used techniques for the characterization of protein structures. While infrared (IR) absorption is active for vibrations that alter the dipole moment, Raman scattering is active for vibrations that alter polarizability [25]. For molecules with a well-defined specific symmetry, such as water, two-atomic gases, or benzene, mutual exclusion rules can be derived based on the properties of their respective symmetry point group. In such a case, Raman responds only to inversion-symmetric vibrational modes, while IR only responds to inversion-antisymmetric modes so that the same band can never be observed in both spectra, a phenomenon known as the mutual exclusion rule [26]. While this is not the case for non-centrosymmetric molecules, such as amyloid aggregates, the relative magnitudes of various vibrational bands are still substantially different between the two techniques and may also be affected differently by conformational changes. Hence different selection rules apply to the two techniques, which therefore provide complementary information of molecular vibrations, as weak IR bands may experience a strong Raman response and vice versa [25]. Other widely used spectroscopic methods for protein structure analysis include nuclear magnetic resonance (NMR), fluorescence spectroscopy (FS), UV–Vis spectroscopy, and circular dichroism (CD). NMR measures the energy required to change the alignment of magnetic nuclear spins in a magnetic field, which depends on the local environments of the atom. NMR is a high-resolution technique and a very powerful tool, as it is able to provide site-specific information on localized segments of the polypeptide chain [27]. FS uses electromagnetic light to excite electrons in the studied samples and measure the subsequent emission of photons when the excited electrons transit back to the ground state via intermediate states [28]. Many native protein sequences contain intrinsic fluorophores—for example, tryptophan side chains—which are particularly sensitive to changes in the local environment, thus providing useful information about tertiary structure [29]. Alternatively, non-native fluorophores can be site-specifically introduced into protein molecules [28]. UV–Vis spectroscopy measures the absorbance of electromagnetic light by the protein’s fluorophores [30], and CD spectroscopy detects differences in absorption by chiral molecules of left- and right-handed circularly polarized light [31]. 

Among the aforementioned spectroscopic methods, NMR is the only one able to provide structural information with atomic resolution. Nonetheless, solid-state NMR is an inherently insensitive technique that requires frozen or lyophilized samples, which must also be isotopically labeled [32]. Solution NMR, on the other hand, can resolve the three-dimensional structure of proteins and provide information about dynamics and intermolecular interactions under physiological conditions [33]. However, solution NMR requires large quantities of pure samples (0.1–1.0 mM) [34] in a soluble form at room temperature and for them to remain stable for the duration of data acquisition, which could take as long as tens of hours [29]. The proteins need to be of small sizes (≤100 kDa) [35] due to the difficulty in understanding the link between chemical shifts and structural parameters [36]. Nevertheless, major contributions to resolving amyloid structures, particularly fibril structure, have been made using NMR spectroscopic techniques, which lie outside of the scope of this review. There exists an abundance of excellent literature covering this topic, to which the reader is referred [34,37,38,39,40,41].

UV–Vis spectroscopy and CD spectroscopy are commonly used in laboratories due to their relatively easy operation and fast data analysis [42]. UV–Vis spectroscopy is very sensitive to interference by stray light or light scattering and suffers from low resolution and overlapping spectral peaks. While UV–Vis spectroscopy can only be used to observe tertiary structural changes of proteins, circular dichroism can detect both secondary and tertiary protein structures, but estimations of the secondary structure composition of CD spectra are less reliable for α/β-mixed or β-sheet-rich proteins, such as amyloid oligomers and fibrils, due to their spectral variability and lower spectral amplitudes [31,43]. FTIR spectroscopy, on the other hand, resolves β-sheet composition with accuracy, which makes it a suitable candidate for the study of amyloids [44]. A major drawback, though, is the strong absorption of IR radiation by water, which overlaps with the amide I band and therefore limits its use for biological samples in aqueous environments [45]. Strategies for overcoming this issue include measurements in deuterated solutions and a method called attenuated total reflection (ATR) FTIR, in which an evanescent IR wave penetrates only a few micrometers of sample layers deposited on the surface of a high-refractive-index material [46]. Alternatively, Raman spectroscopy has a major advantage over IR in that water bands are weak in Raman, enabling the measurement of samples in an aqueous environment close to physiological conditions. In FTIR, the amide I band is primarily used to assign secondary structures to proteins, while in Raman both the amide I and III bands provide structural information. Additionally, Raman provides information on aromatic residues in the region below 1620 cm^−1^ [36]. However, protein signals are usually very weak in bulk Raman acquisition, and therefore, high sample concentrations in the millimolar range are required [47]. To overcome this issue, numerous strategies have been developed to increase the sensitivity of Raman spectroscopy, such as resonance Raman spectroscopy (RRS) and plasmon-enhanced Raman spectroscopy (PERS). The latter is an umbrella term introduced by Ding et al. [48] generalizing the concept of surface-enhanced Raman spectroscopy (SERS) and tip-enhanced Raman spectroscopy (TERS) to include any techniques that exploit the antenna effect in the vicinity of suitably nanostructured plasmonic material interfaces to achieve a highly localized ultrahigh Raman sensitivity. In RRS, an excitation wavelength within the absorption band of the sample is used to enhance vibrational signatures strongly coupled with a small subset of vibrations localized on the resonant chromophore [49]. In contrast to non-resonance Raman spectroscopy, in which all of a molecule’s vibrations contribute to the spectrum with comparable intensities, this results in increased selectivity and enhancement factors as high as 10^8^ [49]. Plasmon-enhanced Raman techniques can provide signal enhancements of up to 14 orders of magnitude compared to conventional bulk Raman spectroscopy [50,51], allowing structural studies down to the single-molecule level. Furthermore, the combination with near-field techniques can enable a great enhancement of the spatial resolution of IR and Raman spectroscopy [52,53].

Other frequently used methods for single-molecule detection are based on fluorescence spectroscopy, such as single-molecule Förster resonance energy transfer (smFRET) and fluorescence correlation spectroscopy (FCS) [54,55]. The challenge lies in the fact that since even tryptophan, the natural amino acid with the highest fluorescence quantum yield (~0.13), is not suitable for single-molecule detection due to its low photostability, labelling with extrinsic fluorophores is unavoidable for single-molecule detection [54]. Fluorescent tags have been shown to significantly modify the size distributions of amyloid oligomers, indicating an impact on their formation, whereas the structures of fibrils remained almost unaffected [56]. Therefore, label-free techniques are preferred for the analysis of amyloid oligomer structure. Table 1 summarizes and compares relevant properties of the aforementioned spectroscopic methods.

## 3. Ensemble-Averaged Studies of Amyloid Oligomer Species

### 3.1. Infrared Spectroscopy

**Figure 2:**
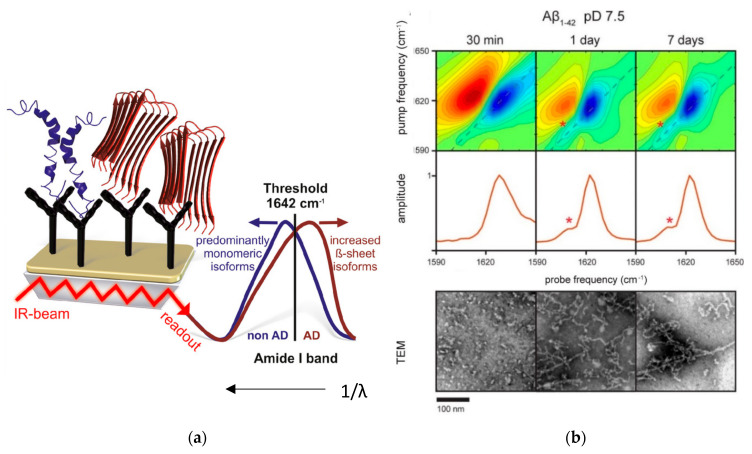
(**a**) Schematic overview of the immuno-infrared-sensor. If the marker band (amide I) is dominated by disordered or α-helical monomeric isoforms, the patient is diagnosed as non-AD (blue). If β-sheet isoforms are enriched (red), the amide I signal is shifted below the threshold (1642 cm^−1^), indicating AD. Part of figure reproduced from Ref. [64] with permission. (**b**) Two-dimensional IR spectra of Aβ42 aggregated for 30 min, 1 day, and 7 days (representative for the transition from oligomers to mature fibrils); diagonal slices through the fundamental transition (dashed line); and representative TEM images for Aβ42. The 1610 cm^−1^ transition is marked with an asterisk in the 2D spectra and diagonal cuts. Part of figure adapted with permission from Ref. [65]. Copyright 2018 American Chemical Society.

The amide I infrared band of proteins is highly sensitive to secondary structure signatures, but strong overlapping water absorptions prevent studies in aqueous environments, leading to the widespread use of deuterated solutions. However, D_2_O is non-physiological, thus raising fundamental questions regarding the impact of isotope exchange on protein dynamics. An alternative way to overcome the problem of strong IR absorption by aqueous solutions is ATR-FTIR. In a recent publication by Milosevic et al. [60], ATR-FTIR was utilized to study the conformational changes during the fibrillization of hen egg white lysozyme (HEWL) over a time period of 0 to 60 days. The native form was measured in H_2_O solution and in 90% ethanol (C_2_H_5_OH) solution. Significant shifts in the amide I and amide III regions of the IR spectra towards β-sheets upon transition to ethanol solution were observed. Deconvolution of the amide I region revealed that the oligomer state is structurally closer to the mature fibril than to the native monomer. To further increase sensitivity and selectivity of this technique, the surface of the internal reflection element can be functionalized with amyloid specific antibodies, such as monoclonal 1E8 antibody for capturing Aβ [61] or monoclonal HT7 antibody for tau detection [62]. Gerwert and co-workers [61,63,64] developed an ATR-FTIR based immuno-sensor for the detection and secondary structure analysis of small amounts of Aβ peptides in their physiological aqueous environment (Figure 2a). In this work, it was demonstrated that this sensor can measure the Aβ peptide secondary structure distribution in the cerebrospinal fluid (CSF) and blood plasma of Alzheimer’s disease (AD) patients. It was found that the amide I band frequency of Aβ peptides, which indicates β-sheet conformation, was significantly shifted in AD patients compared to control patients and thus could be used as a spectral biomarker for AD. 

Two-dimensional IR (2D IR) spectroscopy is another method that was used to discriminate the structure of amyloids [65,66,67]. Advantages of 2D IR over ATR-IR or other linear IR techniques include its ability to measure cross peaks and diagonal anharmonicities that are sensitive to the structure and coupling between different vibrational modes [68,69]. In addition, 2D IR signals scale nonlinearly with the optical field amplitude, resulting in narrower line widths and improved spectral resolution [69,70]. For instance, Lomont et al. [65] utilized this advantage to identify a band at 1610 cm^−1^ in Aβ40 and Aβ42 fibrils (Figure 2b), which does not appear in β-sheet-rich oligomers and cannot be resolved in linear IR spectroscopy because it is covered by the broad characteristic β-sheet band for amyloid fibrils, centered at 1625 cm^−1^. They hypothesized that this 1610 cm^−1^ transition stems from a delocalized amide I mode, and, through spectral modeling of amyloid structures, came to the conclusion that the lower transition frequency of this mode could be explained by differences in β-sheet structure of different regions of the fibril. 

FTIR can further be combined with isotope labeling, where the residue of interest is replaced with an analogue bearing an isotope-labeled ^13^C=^16^O or ^13^C=^18^O carboxylic group, which causes a shift in the amide I frequency. Isotope labeling thus acts as a probe to obtain residue-specific structural information and enables the study of the kinetics of the structural transitions of a mixture of different peptides, as demonstrated in recent studies on Aβ40 and post-translationally pyroglutamylated Aβ (pEAβ3-40) [71] as well as mixtures of Aβ40 and Aβ42 monomers and oligomers [72,73]. Similarly to the aforementioned ATR-FTIR based immunosensor by Gerwert et al., this technique can be further combined with antibody functionalization, as demonstrated by Zanni and co-workers [74]. Their recently developed 2D IR spectroscopic immunosensor combined with isotope labeling was able to capture hIAPP oligomers and distinguish two different fibrillar polymorphs by their structure. These results support the hypothesis that the fibrillar polymorphs emerge from a common intermediate oligomer.

The above-mentioned works represent selected examples of recent studies for the application of IR-based methods used for the conformational analysis of amyloid intermediates. For a more detailed review on ATR-FTIR spectroscopic and 2D IR spectroscopic techniques coupled with isotope labeling which have been used in the past for amyloid conformation studies, the reader is referred to the publications by Li et al. [75] and Sarroukh et al. [76].

### 3.2. Raman Spectroscopy

In recent years, thanks to advancements in the fabrication of nanostructures and nanoparticles, PERS has become an increasingly popular tool for investigating protein structure due to its high spatial resolution and sensitivity. This technique requires a plasmonic substrate material, usually enhanced by a favorable morphology, such as randomly roughened surfaces, individual nanoparticles, and ordered nanostructure arrays [77]. Many studies use gold or silver nanoparticles as plasmonic material. For instance, D’Urso et al. [78] exploited the enhancement properties of silver nanoparticles for the study of hIAPP and Aβ40 oligomers as well as the equimolar mixture of both peptides during their self-assembling processes in aqueous solution at nanomolar concentrations. Analysis of the amide I bands revealed that hIAPP oligomers are rich in β-sheet secondary structures, whereas Aβ40 is rich in α-helices. The spectra of oligomers formed by the equimolar mixture of hIAPP and Aβ40, on the other hand, only contained a shoulder of α-helices as secondary structure. 

Bhowmik et al. [79] introduced a new approach to mimicking cell membranes by coating their silver nanoparticles with a lipid bilayer to study the conformation of membrane-bound Aβ40 oligomers. Hereby, an enhancement in SERS can only be expected if the oligomer penetrates the membrane and draws close to the nanoparticle surface. Additional isotope labeling enabled the secondary structure analysis at the level of individual residues. For the membrane-attached oligomers, a β-sheet–β-turn–β-sheet motif was observed, which is also a shared characteristic of a class of transmembrane pores called “porins” [80]. Therefore, these results support the hypothesis that Aβ oligomers exert their toxicity by forming an unregulated ion channel or “pore” [81], leading to ionic dyshomeostasis across the neuronal membrane with subsequent neuronal malfunction and neuronal death [79].

**Figure 3:**
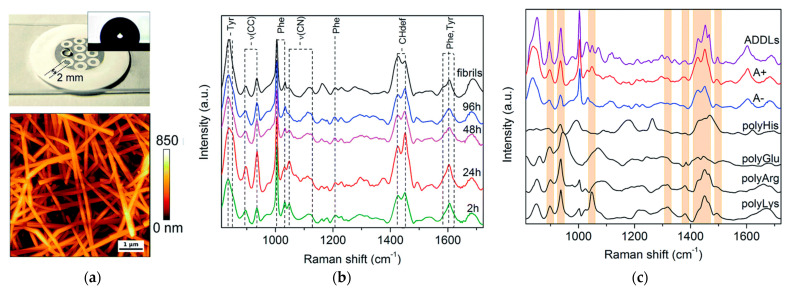
(**a**) Picture of the silver-spotted substrate used for SERS analysis showing a drop of Aβ42 solution deposited on a 2 mm large spot. Inset: contact angle image of a water droplet after deposition on the spot; exemplary AFM image of the spot showing intertwined AgNWs. (**b**) Series of SERS spectra of Ab42 oligomers over 2 h, 24 h, 48 h, and 96 h incubation time and of mature fibrils (from bottom to top). (**c**) SERS spectrum of ADDLs compared to that of type A+ (toxic) and A- (non-toxic) oligomers. SERS spectra of polyHis, polyGlu, polyArg, and polyLys are also displayed for comparison. Bands of polyLys and/or polyArg describing relevant spectral features of type A+ oligomers and ADDLs are identified with colored boxes. Figure adapted from Ref. [82] with permission.

In a more recent study, Banchelli et al. [82] utilized silver nanowires for the detection of Aβ42 intermediates at different stages of aggregation (Figure 3). The Raman spectra showed high similarity between amyloid-beta-derived diffusible ligands (ADDLs), which are known for their high toxicity, and another toxic Aβ42 oligomeric species (A+), whereas differences with respect to those of non-toxic Aβ42 oligomers (A-) were apparent (Figure 3c). Furthermore, those spectra were compared to those of polyLys, polyArg, polyHis, and polyGlu in order to study the specific contribution of side groups mainly responsible for SERS signals characterizing the toxic type A+ and ADDLs forms. From these observations, it was concluded that Tyr, appearing as intense bands at 830, 850, and 1604 cm^−1^, as well as Lys and Arg residues, may be part of the characteristic “toxic” molecular fingerprint of type A+ oligomers and ADDLs. As possible toxicity mechanisms, it was proposed that Tyr residues may facilitate the association of biological membranes with misfolded oligomers when they are exposed on the oligomer surface due to their hydrophobicity, whereas positively charged Lys residues on the oligomer surface may promote the interaction with the negatively charged ganglioside, with further insertion of the hydrophobic oligomer within the membrane bilayer [82]. 

For further applications of Raman spectroscopy to neurodegenerative diseases, the reader is referred to the detailed review by Devitt et al. [83].

## 4. Single-Molecule and Low-Copy Number Studies of Amyloid Oligomer Species

In this context, the term “single-molecule” refers to studies with a resolution of a single aggregate, which is composed of many monomers, thus generally representing a larger scale than the single-molecule limit of non-aggregating biomolecules. This enables the use of techniques such as AFM, which may otherwise be unable to resolve single molecules of smaller species. In addition to this, some techniques do not have the capability to confirm whether a single oligomer or a small number of individual aggregates were detected, which is why we include the term “low-copy-number”, referring to a number of about 10 or less, at which individual signatures appear more prominently than the ensemble average. This transitional regime of low-number statistical data analysis has its own benefits, as briefly discussed in the concluding remarks.

Infrared and Raman spectroscopies have been employed to perform single-molecule experiments of amyloid oligomers. Despite the vast success of Raman spectroscopy in single-molecule experiments in general, it is outweighed by IR in terms of the number of publications to date in the case of amyloid oligomers.

### 4.1. Infrared Spectroscopy

Although Hoffmann et al. [84] still fell short of achieving single-molecule resolution, they probably were the first case of an amyloid oligomer IR-spectroscopic study to come close, doing so by applying gas-phase multiple photon dissociation IR spectroscopy. Without the nano-antenna enhancement of which later works took advantage, it was necessary to employ this nonlinear method, which yields less quantifiable results, in order to achieve the required sensitivity to detect low-copy-number of molecules. This technique was used in tandem with THT staining, TEM and ion mobility-mass spectrometry to characterize various aggregation states of NFGAIL, an amyloidogenic model peptide. With this method, the authors were able to associate oligomers with compact and extended morphology with β-turns and β-sheets, respectively.

**Figure 4:**
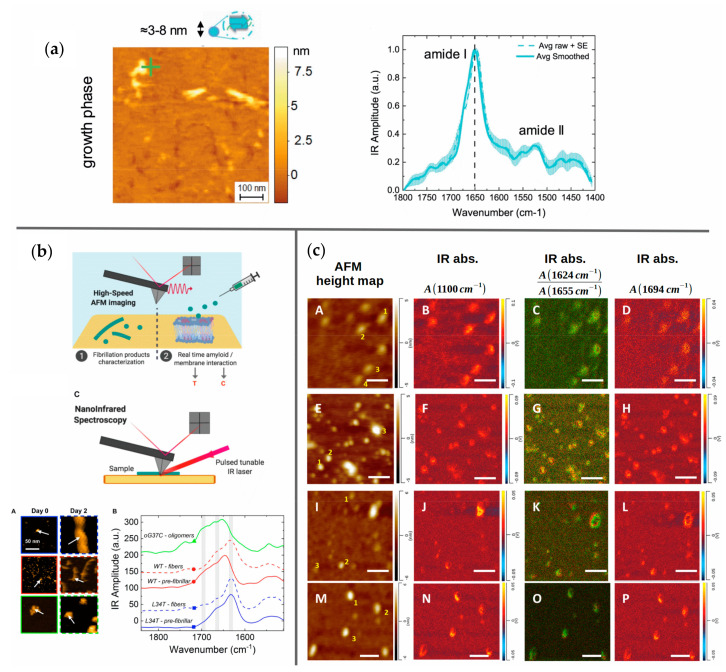
(**a**) AFM maps of Aβ42 oligomers with corresponding IR spectra. Part of figure reproduced from Ref. [88] with permission. (**b**) Schematic illustration of an AFM-IR setup (above). AFM maps of amyloid fibrils and oligomers with corresponding IR spectra (below). Part of figure reproduced from Ref. [89] with permission. Copyright 2020 Feuillie et al. (**c**) AFM maps and corresponding IR line absorption maps of various oligomeric aggregates. Part of figure adapted with permission from Ref. [90]. Copyright 2021 American Chemical Society.

Ruggeri and co-workers successfully studied amyloids in various aggregation states at single-molecule resolution using AFM-IR, demonstrating the capability of simultaneous data acquisition by two fundamentally distinct techniques at the single-molecule level (Figure 4a). To this end, a microdroplet sample deposition technique was developed [85] and the AFM setup parameters were optimized [86]. A tunable quantum cascade laser was integrated in the system to perform off-resonance low-power short-pulse infrared nanospectroscopy [87]. The IR absorption amplitude is determined with the AFM tip itself by thermochemical detection, removing the need for using an optical spectrometer. On this platform, Aβ42 was studied [88], matching up morphological and elastic properties measured by AFM with amide I signatures of the secondary structure to infer a change from anti-parallel to parallel β-sheet stacking structure in the transition from oligomeric to fibrillar species.

Feuillie et al. [89] investigated wild-type Aβ42 and two mutated variants, L34T and oG37C, in the presence and absence of a model lipid membrane. In Figure 4b, a schematic depiction of the AFM-IR setup is shown, as well as AFM maps of amyloid fibrils and oligomers with corresponding IR spectra. We focus here on the spectroscopic aspects of the presented data, for the very detailed AFM-related results, the reader may refer to the original publication. The IR amide I signature revealed that the rapidly aggregating L34T variant’s secondary structure was dominated by parallel β-sheets as early as one day after incubation was initiated. In contrast, the oG37C variant, which is known to form no fibrils even after long incubation times, showed strong β-turn and anti-parallel β-sheet signatures. Interestingly, Aβ wild type exhibited a mixed signature of β-turns together with parallel and antiparallel beta sheet stacks at intermediate incubation times before shifting towards a parallel beta-sheet-dominated signal, very similar to L34T. The authors concluded that the wild type initially engages in various aggregation pathways leading to both parallel and anti-parallel β-sheet structures, and subsequently undergoes a slow process of structural reorganization into parallel β-sheet-dominated fibrils. Additionally, the interaction of the three Aβ variants with a model membrane was observed in operando. The (non-single-molecule) IR signatures after 4 h of incubation time revealed a presence of anti-parallel β-sheet rich structures embedded in the oG37C-injected and, to a lesser extent, the wild-type-injected membrane but not in the sample with the L34T variant. The work features a detailed discussion of aggregate structures with numerous literature references.

Dou et al. [90] employed AFM-IR to study the aggregation process of α-synuclein in the presence of two different phospholipids. A significant influence of added phospholipids on the formation of α-helices, parallel and antiparallel β-sheets was reported. In this work, the scanning capability of the system was exploited by creating 2D maps of IR absorption amplitudes that can be correlated to AFM height images. For this, the IR absorption for selected wavenumbers was recorded in order to display the presence of phospholipids, the ratio of parallel β-sheets to α-helix, and the presence of antiparallel β-sheets, respectively, in separate maps (Figure 4c). In contrast to other works, spectra from several sample points for each condition were shown, giving the reader an impression of the natural variability of single-molecule results. Overall, this work stands out in terms of utilizing and visualizing the IR spectral data in a more effective way than others.

Waeytens et al. [91] studied Aβ fibrils and oligomers by AFM-IR, reporting a detrimental effect of ZnSe substrate surface on the sample, drawing no further conclusion about the structural composition of the studied aggregates. Banerjee et al. investigated aggregates of tau [92] and Aβ42 [93] by AFM-IR. While tau oligomers were not discussed in the main text, tau fibrils as well as Aβ42 oligomers and fibrils were reported to exhibit unexpected β-structure signatures in some cases, indicating a significant variability in structure on the single-molecule level, in contrast to common presumption.

### 4.2. Raman Spectroscopy

Various variants of PERS have been extensively applied to single molecule studies for two and a half decades [48,94,95,96,97,98,99]. Continuous advances in terms of understanding and optimizing the material and morphological properties of plasmonic nanoantennas [51,100] have further increased sensitivity and accuracy, as exemplified by the following studies. TERS, in a similar fashion to AFM-IR, combines plasmon-enhanced Raman spectroscopy [48] with AFM. Already for a number of years, a wealth of work has been published on single-molecule TERS of amyloid fibrils [101,102,103,104,105,106,107]. We would like to highlight the work by Deckert-Gaudig et al. [108], in which the hydrophobicity of insulin fibril subdomains was mapped and co-localized with amino acid TERS signatures at a high spatial resolution.

**Figure 5:**
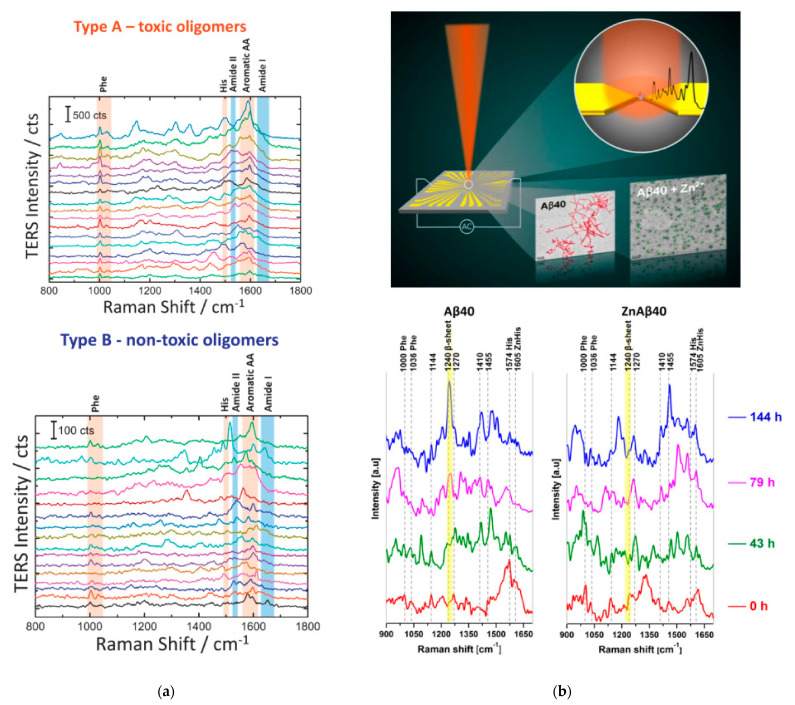
(**a**) TERS spectra of toxic and non-toxic oligomer samples. Part of figure reproduced from Ref. [111] with permission. Copyright 2018 WILEY-VCH Verlag GmbH & Co. KGaA, Weinheim. (**b**) Above: Cartoon of the working principle of the nanogap device. Peptides are trapped via DEP inside the gap between a pair of electrodes and the SERS signal is simultaneously measured (upper inset); TEM images of Aβ40 aggregates with and without the presence of Zn^2+^ (lower insets); below: SERS spectra of Aβ40 in solution trapped with the nanogap device with and without Zn^2+^ after different incubation times. Figure adapted with permission from Ref. [112]. Copyright 2021 American Chemical Society.

Not many publications could be found related to PERS of oligomeric amyloid species. Bonhommeau et al. [109] also used the wild-type and L34T/oG37C variants of Aβ42, demonstrating the capability to distinguish between the final stages (one month incubation) of the three different variants (two in fibrillar, one in oligomeric form) by monitoring the amide I and III bands. Devitt et al. [110] demonstrated differentiation of distinct intermediate and final aggregation stages of BSA, β2-microglobulin and tau proteins based on a number of Raman modes from the amide I region and aromatic amino acids after principal component analysis of the spectra. Since the aim of these papers was to demonstrate the distinction capability, no further discussions of structural implications of the spectral features were presented. D’Andrea et al. [111] used a TERS setup to investigate oligomers of HypF-N, an amyloidogenic protein extracted from E. coli in the single-aggregate regime (TERS spectra shown in Figure 5a). In contrast to other SERS and TERS experiments, the analysis of the amide III band was dropped in favor of the amide I region. A distinction between toxic and non-toxic oligomers was achieved by performing a Lorentzian fit decomposition of spectral contributions of the amide bands and aromatic amino acids. A discussion about possible implications of amino acid conformational changes with regards to interactions between oligomers and the cell membrane distinguishes this work from the rest.

The setup used by Vu et al. [112] differs significantly from the TERS and IR assays discussed above in that the measurements can be performed in the solution phase since no AFM is involved. Instead, a pair of Ti/Au electrodes with a nanogap that acts as plasmonic nano-antenna is used to trap a low copy-number or single molecule of amyloid protein via dielectrophoresis (DEP) directly in the SERS hotspot and is coupled with a confocal Raman microscope for SERS measurements (Figure 5b). While single-molecule detection cannot be confirmed with direct evidence on this platform, the application of DEP trapping at such low target concentrations strongly suggests that at least some of the spectra taken in solution phase were recorded from single molecules [96,113]. Aβ40 was studied in the absence and presence of Zn^2+^ ions after four different incubation times from 0 h up to 144 h, with spectra taken in solution, but also in the dried phase, which helped reveal features in the amide I region that were absent in solution. In addition to assigning secondary structures to the various aggregates, Phe and His signatures were analyzed, and their structural implications were discussed.

## 5. Discussion

The single-molecule resolution of AFM-IR was demonstrated to be a valuable tool for obtaining conclusive results regarding the structure of heterogeneous intermediate amyloid protein aggregate stages. Specifically, the use of a tunable laser source and the instant detection method enable fast scanning of a large area at a specific excitation wavelength. Since most of the time, the interest is focused on a few signature wavenumbers in the amide I region, this opens up possibilities to observe a time-resolved series of such specific signature in a 2D-mapped region, as demonstrated by Dou et al. [90]. However, there is still room for improvement in this new and promising technique. For instance, the system works with a gold-coated AFM tip over a gold surface, inducing a nano-antenna effect [87], which, similar to TERS, is potentially subject to a plasmonic nanogap enhancement in the relevant IR range [114,115,116]. It appears that this facet of AFM-IR has not been discussed in the literature to date. However, there may be an opportunity to optimize the spectroscopic performance by adjusting the plasmonic response by various methods, as exemplified by PERS and subsidiary fields (see the above section for relevant references). Overall, the spectroscopic aspect of the experiment was in some cases not exploited to its full possibilities. By highlighting the works that made excellent use of the technique, we hope that our readers may take inspiration to make the best of their experiments. Regarding oligomer samples, due to individual variations, the correlation of aggregate species and secondary structure signature may not always be obvious on the single-molecule level. Care should be taken when inferring general conclusions from limited data sets. Statistical significance is a key factor in single molecule experiments, especially when drawing comparisons to ensemble averages.

### 5.1. Comparing Various Single-Molecule Methods

Most of the works reviewed in this section took advantage of the highly localized nano-antenna resonance. At this point, there is probably no other technique that can match its ability to focus a greatly enhanced optical field within a nano-scaled volume in dry or liquid conditions that can be easily coupled with other techniques and operated with commercially available optical equipment, especially if the surface plasmon response is well-matched with the excitation wavelength. The recent application to IR spectroscopy shows its great versatility, with expectation that it can be applied to a greater variety of optical techniques in the future. The main strength of both AFM-IR and TERS lies in the simultaneous structural investigation at the single-molecule level with a combination of two powerful techniques yielding correlated morphological, mechanical, and vibrational information of the same entity. A major constraint of both techniques is the need to operate in dried conditions, which is underlined by the efforts that are made to mitigate adverse effects of the drying process and to confirm that certain structural features are preserved after drying. In direct comparison, the main drawback of AFM-IR as opposed to TERS is the need to scan the spectral range, limiting the acquisition rate for recording wide-range spectra. Ruggeri et al. [87] mentioned a scan rate of 100 cm^−1^ s^−1^. On the other hand, if one is only interested in one or few specific spectral bands, AFM-IR may be the better option since it can achieve higher acquisition rates when applied to a very limited spectral range. Apart from that, IR and Raman signatures complement each other, so in the future, one may explore the possibility of integrating them into the same system, thus enjoying the joint merits of both.

Of the works reviewed in this section, only Vu at al. measured vibrational spectra in aqueous solution in the quasi-single-molecule regime. While secondary structure features have been proven to be still detectable in dried samples, it cannot be assured that the structure is not affected otherwise. For that reason, it is preferential to perform single-molecule experiments of oligomers in the solution phase, especially when investigating less explored details of the spectrum. An approach to solving this problem was presented by Khatib et al. [52] and Lu et al. [117], who demonstrated nano-scale IR spectroscopy of liquid samples. The problem of IR absorption is circumvented by encapsulating a very small water volume in between graphene monolayer sheets and detecting scattered light rather than transmitted light. This technique belongs to the family of scanning near-field optical microscopy (SNOM) and also takes advantage of the plasmonic nanoantenna effect. While, to the best of our knowledge, no single-molecule results have been published with this method, the potential to reach single molecule resolution has been proven in dry environments for IR and Raman variants of SNOM [53]. In particular, Paulite et al. [118] studied single amyloid fibrils by IR-SNOM, indicating that with some further development in the sensitivity, the goal may come within reach to investigate amyloid oligomers in liquid by IR spectroscopy. The spatial isolation of a low number of amyloid aggregates and/or monomers, which may be adjusted by dilution, in a very small water volume offers an advantage in freezing the aggregation process—especially for smaller intermediate aggregates—as in bulk conditions, the aggregation tends to continue during the experiment. It would be beneficial if this method could be applied to other branches of nanospectroscopy, such as TERS or AFM-IR, although the high lateral thermal conductivity of graphene [119] as well as thermal dissipation in water must be considered when trying to apply it to the thermochemical detection of single molecules in AFM-IR. Alternatively, other IR detection methods with single-molecule capability could be explored for the use with AFM in order to overcome the issue.

When using TERS, there is still much potential to develop this powerful technique, such as by venturing further into the lesser-known waters of non-amide Raman bands, as demonstrated by Deckert-Gaudig et al. [108], using the AFM setup to create linescans (such as Deckert-Gaudig and van den Akker [102] did for fibrils) or 2D maps based on Raman signatures, or by analyzing time-lapse series of Raman spectra. Finot et al. [120,121] demonstrated the use of statistical data to infer correlation of different amino acid signals as an example for structural information beyond α-helix/β-sheet/random coil structure that can be extracted from spectroscopic data. Raman spectra can easily be acquired at rates around one scan per second. For instance, Huang et al. [122] measured low-copy-number signals of amino acids with an exposure time of 0.1 s. Similar to Vu et al., a dielectric force related to DEP was applied to guide the target towards a SERS hotspot. Dielectric force offers a very reliable and low-destruction trapping technique [123] suitable for single-molecule label-free PERS, since proteins typically can be trapped readily by such methods [123].

### 5.2. Impact

Key questions regarding the disease, such as environmental influence on aggregation pathways or drug efficiency, can be addressed by analyzing the amide I or III region of single spectra, as demonstrated in the reviewed works. The quick and reliable differentiation of various toxic and non-toxic oligomer species as well as fibrils gives a promising prospect for high-throughput screening of potential drugs or diagnostic substances. However, there is much room to further exploit the spectroscopic data obtained from such sensitive experiments. Often, much time and effort are put into the development of novel high-end platforms; while outstanding sensitivity is demonstrated with standard samples, much less frequently is this followed up by a systematic application to targets of practical interest. Despite this, all the authors reviewed in this work have embraced the challenge to depart from the well-trodden paths and venture into directions that benefit the advance of medical science. Hopefully, in the future, more attention can be drawn into the study of impactful pathogens, such as amyloid oligomers, at the single-molecule level.

## 6. Concluding Remarks and Perspectives

In the reviewed works, the powerful spectroscopic techniques often appear to play a minor supporting role in the analysis, and the results fall short of advancing our fundamental understanding of the various aggregation pathways of amyloidogenic proteins by elucidating new details about oligomer structure. We hope that these outstanding techniques can be used more frequently to observe the natural state of single molecules, even at the risk of not being able to explain all observations.

The mechanism leading to different amyloid aggregation pathways is still poorly understood. This is mostly due to non-fibrillar oligomers being a group of high diversity [84], often lacking long-term stability, that cannot be studied in detail in large ensembles. However, this diversity also presents itself as a fascinating opportunity to study a variety of conformations assumed by assemblies of the same basic building blocks and in this way unlock our understanding of a variety of medical conditions. The key idea of performing single or low-copy-number molecule studies is to avoid errors and missed information caused by ensemble averaging. The opportunity to observe a multitude of fluctuating properties that tend to get obscured in averaged measurements easily gets hampered when trying to improve the repeatability of the experiment, for example, by deliberately fixing the sample in place, suppressing dynamic conformational changes or time-averaging recorded spectra.

Single molecule techniques are best exploited in a relatively low-signal-to-noise regime. Molecular conformational changes and Brownian motion may affect position and width of spectral signatures on short timescales. This means that by evaluating a large enough amount of statistical data with short exposure time, more diverse information may be revealed that otherwise would remain veiled in spectra averaged over an ensemble or a long exposure time. The spirit of this principle is exemplified by well-established low-copy-number techniques, such as dynamic light scattering or fluorescence correlation spectroscopy, and the above-mentioned recent approaches to time-lapse spectral analysis.

## Figures and Tables

**Table 1:** Comparison between various spectroscopic techniques.

	Fluorescence	NMR	CD	UV–Vis	FTIR	Raman
**Basic principle**	Light emission by residual aromatic amino acids	Nuclear spin relaxation	Differential absorption of circular polarized light	Electronic transitions	Vibrations of molecular bonds (changes in dipole moments)	Vibrations of molecular bonds (changes in polarizability)
**Resolution**	Medium (tertiary structure on a local level)	High (secondary and tertiary structure on a global and local level)	Low to medium (secondary and tertiary structure on a global level)	Low to medium (tertiary structure on a global level)	Low to medium (secondary structure on a global level; tertiary structure on a local level with isotope-labeling)	Medium to high (secondary and tertiary structure on a global level)
**Sensitivity**	Single molecule (extrinsic FS)–μM (intrinsic FS)	0.1–1 mM	μM–mM	μM	0.1–1 mM (proteins), 1–100 mM (small molecules)	Single molecule (PERS)–mM (bulk Raman)
**Limitations**	Photostability issues, limited fluorophore lifespan, auto-fluorescence; fluorescent labeling might affect protein aggregation and structure (extrinsic FS)	High sample purity, sample size limit ≤100 kDa (solution NMR); high amount of sample, lyophilized and isotopically labeled samples (ssNMR)	Less accurate predictions for β-structure than for α-helices	Stray light and light scattering interferences, overlapping of spectral peaks	Water interference, overlapping of spectral peaks	Fluorescence interference, photodecomposition and low signal (bulk Raman); requires appropriate substrate/plasmonic structures (PERS)

References: [31,34,35,36,42,43,45,50,51,57,58,59].
